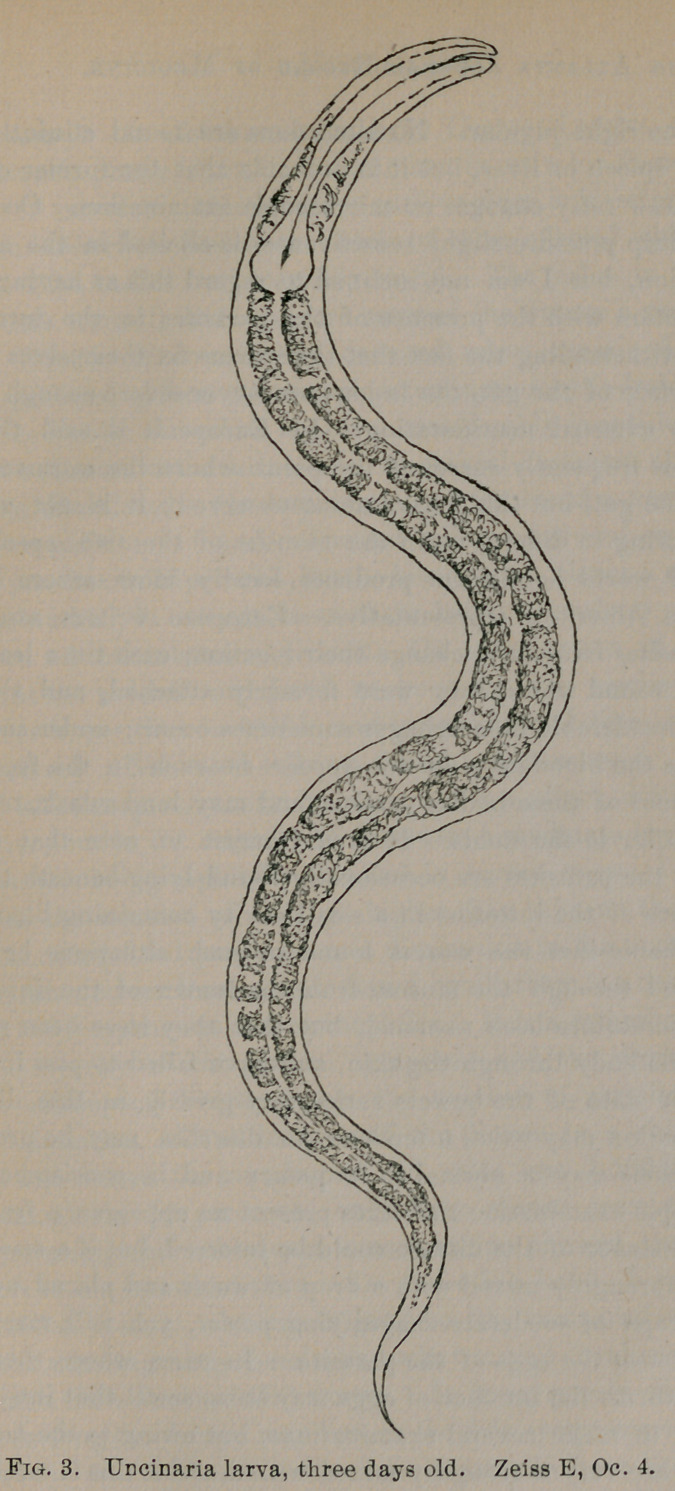# Uncinariasis (Ankylostomiasis); Its Frequency and Importance in the Southern States

**Published:** 1903-06

**Authors:** H. F. Harris

**Affiliations:** Atlanta, Ga.


ATLANTA

### Journal-Record of Medicine.

Successor to Atlanta Medical and Surgical Journal, Established 1855, and Southern Medical Record, Established 1870.

VOL. V. JUNE, 1903. NO. 3.

BERNARD WOLFF, M.D., EDITOR, Nos. 319–20 Prudential.

Published Monthly.

M. B. HUTCHINS, M.D., BUSINESS MANAGER, No. 64 Marietta St.

### ORIGINAL COMMUNICATIONS.

### UNCINARIASIS (ANKYLOSTOMIASIS); ITS FREQUENCY AND IMPORTANCE IN THE SOUTHERN STATES.*

BY H. F. HARRIS, M.D.,

ATLANTA, GA.

Uncinariasis, or hookworm disease, has long been known as an affection that is occasionally encountered in the United States, but its great frequency and importance is just dawning upon us. In the excellent monograph of Mosler and Peiper on animal parasites, in Nothnagel's "Special Pathology and Therapy," the statement is made that instances of this disease were reported by Duncan in Alabama and Lyell in Georgia in 1849. I regret that I have been unable to verify these references, though there can be no reasonable doubt as to their authenticity. Within recent times quite a number of cases of this disease have been reported from the United

*Read at meeting Georgia Medical Association, Columbus, April 17.

States, though many of the patients were foreigners, and the malady was unquestionably contracted in the old world. Instances of this kind are of comparatively little interest, for the reason that they were not acquired in America, and that in most cases the sufferers lived in the northern part of our country, and the conditions there are rarely such as to permit the distribution of the disease. Interest in this subject was greatly added to by the discovery of Stiles about a year ago that there is a hookworm that is a distinct American species, and to which this writer has given the name Uncinaria Americana. This parasite greatly resembles the one found in the old world, and could easily be mistaken for it, but it exhibits differences that unmistakably stamp it as a new species, and its discovery would point to the probability that the disease is widely disseminated in America, especially in the southern portion of the United States. In Georgia this matter seems to have been entirely dropped after Lyell's work upon the subject, no case apparently having been diagnosed during life until the one that I reported at the last meeting of this Association. At the time that I read my paper I ventured the prediction that this disease was much more common than was generally supposed, but I confess that I had no suspicion of the frightful ravages that it is making among us.

I spent the summer of last year in the mountainous region of northern Georgia, and was much astonished to find that this disease affected a large percentage of the population in many districts, the unfortunate sufferers being generally regarded as dirt-eaters. In September I made a trip through southern Georgia and northern Florida for the purpose of studying malaria, and while there I discovered the fact that almost all instances of profound anemia were due to the uncinaria, and not, as has been heretofore generally assumed, to the malarial parasite. It is a rather curious fact, for which I will later give a possible explanation, that this hookworm disease does not appear to exist to any extent in middle Georgia, and it is further noteworthy that the disease is preeminently one of the country districts. Since beginning my work on this subject I have seen eighteen instances of the disease, and have made the diagnosis in twelve other cases from this State, Alabama and Florida, the feces having been sent to me by various physicians from the localities named. In this connection I wish especially to thank Dr. W. P. Lovvorn, of Cecil, Ga., and Dr. V. O. Harvard, of Arabi, Ga., for their interest and assistance in obtaining feces for examination. Dr. E. D. Bondurant, of Mobile, Ala., who kindly permits me to quote him, informs me in a recent letter that he has within the last year diagnosed seventy-five instances of this malady.

*Natural History of the Uncinaria Americana.*—This parasite belongs to the order of nematodes, or round worms, family strongylidæ, subfamily strongylinæ, and genus uncinaria. The worm is a comparatively small one, the female being always larger than the male. The former varies in length from 11–14.5mm., the average being 13.87; at their thickest portions they are from 0.410–0.600mm., with an average of 0.509mm. The male is from 8–10mm. in length, the average of a large number of measurements being 8.8mm.; at their widest portions they are from 0.300–0.390mm., with a mean of 0.343mm. The worms taper toward either end, and at their cephalic extremities both sexes have about the same diameter, this varying from 0.088–0.132mm. The tail of the female terminates in a pointed extremity, while that of the male ends in an expanded bursa, which at its free end has a considerably greater diameter than the body of the parasite. There are still other peculiarities of the American hookworm that serve to indicate its specific characteristics, but a more accurate description would scarcely be in place on this occasion. I would refer those who wish to study the more minute anatomy of this parasite to the description given by Stiles in his paper last year, which has already been referred to.

The eggs of this hookworm appear to be somewhat larger than those of the old world species, as I have found from a large number of measurements that they vary in length from 57.5 to 80m., and from 35–52.5m. in diameter, the average being 66.52m. and 42.53m., respectively. The eggs, as encountered in the feces, are oval in shape and are covered externally by an extremely thin shell which is perfectly transparent. The solid contents of the eggs do not appear usually to be in contact with the capsule, but there intervenes between the two a clear space that is doubtless filled with fluids. When the eggs are perfectly fresh some of them contain a single granular body within their centers, within which there is a nucleus. In most instances, however, we do not find a solitary body of this kind, but, as a result of rapid multiplication, there are a number of these cell-like structures. At the proper temperature and in the presence of moisture the granular bodies within the eggs rapidly increase in numb er, and in the course of from twenty-four to thirty-six hours completely fill the egg cavity; shortly following this the embryo takes form, and we find in the place of the preexisting structures within the egg a minute snakelike worm which exhibits more or less active movement. Quickly following this the capsule of the egg bursts, and the embryo is liberated, coming out tail first in all instances where I have had an opportunity of observing the process. When the embryo emerges from the egg it has a length of from 350–400m., but in the course of a day or so grows in length from 600–700m., with diameters at their widest portion of from 25–30m. The worm at this stage possesses the power of very active movement, progressing either forward or backward with considerable rapidity and apparently equal ease. Externally it is covered by a transparent chitinous membrane about 5m. in thickness, through which the comparatively simple structure of the internal portion of the parasite may be observed. In a few days after the larva is hatched the body recedes from its outer covering, and a second membrane is formed over the external surface of the worm. Between the outer covering and the second one formed internal to it there is a clear space within which the worm moves. The entire body is still capable of active movement, though this is never so active as before. This is probably the infective stage of the parasite. At a later date many of the worms lose their outer covering, but whether a second one is formed I cannot say with certainty, though it is stated by Loos that this occurs in the old world species. Like the eggs the larvæ die very quickly when dried.

Before quitting this part of the subject it may be as well to call attention to the conditions under which the embryo develops within the egg, as this is extremely important in connection with the dis-

FIG. 1. a, Male uncinaria, natural size; a1, male uncinaria, greatly magnified; b, female uncinaria, natural size; b1, female uncinaria, greatly magnified.

semination of the parasites. In order for the eggs to hatch the temperature must be comparatively high, the development scarcely occurring at all below 70° F. From a number of experiments it appears to me probable that hatching goes on most rapidly between 80° F. and 90° F. In addition to a comparatively high temperature it is necessary that the eggs be moist, they being invariably killed as a result of desiccation; it is, however, true that they will not undergo development if they be covered with water, probably for the reason that oxygen is excluded. The medium in which the eggs hatch best is probably that in which they naturally occur—the feces—though this likewise takes place readily when they are placed in finely powdered sand, or, as has been suggested, in pulverized animal charcoal, both being, of course, properly moistened. The latter substance is extremely favorable for growing the larvæ for laboratory work, inasmuch as the disagreeable odors that arise from the feces are very quickly destroyed. From the foregoing it is seen that the eggs, as we would of course expect, are deposited under conditions that most favor their development, it being assumed that the temperature is favorable. It is, however, true that were the feces allowed to lie undisturbed only the eggs would hatch upon the outer surface where oxygen is present, but it is extremely interesting to note that very quickly following the deposition of the stools eggs of flies are layed upon the feces from which larval forms of these insects are rapidly developed, and they burrow into the masses and break them up to a considerable extent, and in this way oxygen is brought in contact with the ova. It is thus seen that there can be no doubt that flies and other insects that feed upon the feces greatly assist in the spread of this disease. It it of some practical interest to note that the eggs are killed by a freezing temperature, and that if from any cause hatching be prevented they undergo fatty changes and perish in a month or six weeks.

After the larvæ are matured they find their way into the small intestine of the human being, attach themselves to the mucous surface, and, growing rapidly, at the expiration of a few weeks develop into the adult worms. As to how quickly this occurs we are in ignorance so far as the American species is concerned, but the time probably does not materially differ from that which is necessary for a similar process to take place in the old world parasite, and in this case it is known that about six weeks are required. As soon as the worms complete their growth copulation takes place, and the females begin to lay enormous numbers of eggs, after which the cycle of development is begun over again.

FIG. 2. Eggs of the uncinaria, the contents showing varying stages of development. Zeiss E, Oc. 4.

Since it is necessarily true that the larvæ of this parasite in some way reach the human body from the feces in which they are matured, very early in my studies of the malady produced by them I investigated carefully the different ways by which this result might be brought about, and was particularly struck with the fact that the disease occurs preeminently among the poor, and in an overwhelming majority of instances where the matter could be investigated it was found that the sufferers and their families did not use privies, the feces being deposited in and around stables, and oftentimes in the yards. I am much inclined to the opinion that the comparative immunity of the middle Georgia districts from this disease is due to the fact that this habit is not so general, and that almost everywhere water-closets are in use. It is a further fact of importance that the sufferers are in a large majority of instances children who, especially during the summer months, go barefooted. Now there seems to be but two probable explanations of the manner in which the uncinaria larvæ enter the human body; the one—which undoubtedly sometimes occurs—is that the young parasites are swallowed with the food or drink, and the other—which has not as yet been conclusively shown to be true—is that the larvæ penetrate through the skin and, passing through the body, ultimately enter the alimentary canal. If the former were the usual method of infection, adults ought to be quite as often affected as are children, while, as has just been stated, this is not the case. If we accept the latter explanation, we at once understand the relative frequency of the disaase under the circumstances referred to, namely, the constant exposure of the skin of the hands and feet of the children to filth swarming with the uncinaria larvæ. Of course, it is true that the young parasites might be washed by the rains into wells, but it is a fact that as soon as they are placed in water they immediately begin to sink, and in a short time rest on or near the bottom of the vessel in which the fluid is contained, and there is no reasonable doubt that this would occur under the circumstances named. In north Georgia, where the disease is very prevalent, the inhabitants drink spring water exclusively, and it seems highly probable that if the larvæ were washed into the springs they would be almost immediately carried away. Great interest was excited some years ago by the discovery of Loos that the larvæ of these parasites may penetrate the skin of the human being, and although I have myself failed to confirm his observations after a number of experiments, I still feel inclined to the belief that he is correct. It should, however, be stated that this view has recently been combatted by Pieri, working under the direction of Grassi. This writer, Grassi, and another student of the latter, Noe, placed upon the surfaces of their bodies fluids containing larvæ of the old world parasite, and two and a half months later Pieri found eggs of the worms in his feces, but the other experimenters remained free from any evidences of the presence of the worms in their intestines. Notwithstanding that in one out of three instances the experiment apparently proved successful, these investigators concluded that infection in this case probably occurred in some other way, and that the view of Loos is incorrect. In connection with this subject the observations of Bentley are extremely interesting. He showed that among the coolies of India uncinariasis is almost universal, and that ground-itch is so severe and common among them that it has proven a serious hindrance to farming operations. Bentley conceived the idea that the latter condition was due to the penetration into the skin of those so affected with the larval forms of the uncinaria, and succeeded in inducing a disease in every way similar by applying to the bodies of some of the natives earth containing hookworm larvæ. It is thus seen that it seems a possibility that two diseases that hitherto we have not dreamed had any connection with each other may be due in reality to the same cause acting in different portions of the body. In support also of this view it may be remembered that a parasite of the horse, the strongylus armatus, has been shown to penetrate into the intestines of these animals through a similar circuitous route.

*Pathologic Anatomy and Symptomatology.*—The morbid alterations that occur in this disease are so intimately associated with the symptoms that I consider them together in order to avoid much useless repetition. The larvæ of the parasite, when brought in contact with the skin of the human being unquestionably, as Loos has shown, give rise to inflammatory phenomena, and while it cannot be asserted with certainty we have strong reasons for believing that the disease known as ground-itch in English-speaking countries, and pani-ghao in India, is induced in this way. The first manifestations then that the uncinaria probably give rise to are an acute inflammatory condition of the surfaces of the feet, and in rare instances of the hands. This condition may become so severe that walking is painful, and labor requiring this impossible. Following this, the exact period being as yet unknown, the patient begins to suffer from the presence of the parasites in the intestinal tract. We are likewise in ignorance as to the first clinical manifestations that occur in this connection, but we find when the sufferer falls into our hands that he is invariably pale and obviously decidedly anemic. He may complain more or less of digestive disturbances, and he feels a strong disinclination to labor of any kind. The patient oftentimes complains of neuralgic pains, particularly in the head. The appetite may be normal, but more or less anorexia is generally present. It is a curious fact, which appears to be rather characteristic of the disease in the new world that the patients get much better in the winter, and in many cases they assert that they are perfectly well, but as spring advances their strength rapidly declines and by the beginning of summer in severe cases they are in a really pitiable condition. On physical examination it is found that in children growth has taken place with extreme slowness from the very beginning of the disease, it being common to find young people from fifteen to twenty years of age who appear to be from eight to twelve years old. Not only do they present the appearances of youth much greater than is in reality the case, but the growth of the mind and of the various parts of the body is much retarded. This is particularly noticeable in the total want, or only partial development of the beard in the male sex. The skin is found to be thin, wrinkled, and extremely pale, and the mucous membranes exhibit even a greater degree of want of color. The body weight may be somewhat decreased, but as in many other profound anemias emaciation is never pronounced. In severe cases the lower extremities and face are oftentimes found to be swollen, there being occasionally a very pronounced dropsy present. In women the menses are, as a rule, suppressed in severe cases, though this does not prevent the bearing of children. Physical examination of the lungs shows nothing abnormal as a rule, though pleurisy may complicate the disease occasionally. The heart is found in its normal position, and it does not exhibit any enlargement, but we find on auscultation that almost invariably soft blowing murmurs may be heard in the mitral and pulmonary areas; in the tricuspid region murmurs are likewise sometimes encountered, all being evidently hemic. A venous hum is quite constantly heard over the right jugular. No alterations are found clinically in either the spleen or liver, but it is probable that the former organ would show fatty changes on microscopic examination. Occasionally on deep pressure slight sensitiveness is elicited in the abdominal region, but I am not inclined to regard this as having a direct connection with the presence of the parasites in the intestine, for notwithstanding the fact that the worms fix themselves to a mucous surface of the gut, the lesions are never severe enough to give rise to external manifestation. In Europe it is said that petechial spots frequently surround the point where the worms are attached to the gut, but this I have never observed; it is not unlikely that owing to differences in the mouths of the two species that the old world hookworm produces locally more severe lesions than its American representative. European writers assert that the parasites frequently change their location, each time leaving an open wound where they were formerly attached, and that as a result considerable hemorrhages sometimes occur; under such circumstances the blood-cells do not appear as such in the feces, but the pigment of these bodies persists, and may lend a dark, reddish-brown color to the stools. It is of interest to note that on investigation the parasites are occasionally found lying beneath the mucous surface of the intestine in a small cavity containing blood. It seems probable that the worms found in such situations have not penetrated through the mucosa from the lumen of the intestine, as has heretofore been assumed, but that they have been received into the body through the skin, and have failed to pass into the gut. The state of the bowels varies very greatly in this disease; constipation may exist, a tendency to diarrhea may be present, or the stools may be normal in frequency and in consistence. On macroscopic examination the feces present no appearance from which the existence of the disease could be inferred, but if a small particle be thoroughly mixed with a drop of water and placed under a microscope of moderate magnifying power, you will rarely ever fail to detect the eggs of the parasite. In cases where there are but few worms the number of eggs may be so small that it will be necessary to make several examinations, but owing to the fact that the ova are very thoroughly mixed with the feces this is rarely necessary. In addition to the eggs Charcot-Leyden crystals are generally present, but I have searched in vain for them in a number of instances; this is rather curious since it is stated by European authorities that in the stools of those affected with this malady these bodies are invariably found. The urine is in most instances normal, but in severe cases of the disease a small quantity of albumin is occasionally detected when examined for in the proper way, and on microscopic examination cylindroids are sometimes found. In all of my cases considerably quantities of indican were present in the urine, and in several instances the chlorides were diminished. On examination of the blood a very characteristic condition is found. The red cells are reduced anywhere from 10 per cent. to 75 per cent., but the hemoglobin shows a much greater diminution, in severe cases there being sometimes only 10 per cent. of this substance present. On examination of fixed specimens the red cells are found to be sometimes smaller and sometimes larger than in health, and irregularity of form is frequently observed. Nucleated cells are found in severe cases. The number of white corpuscles may be normal, or a slight diminution may occur, but in the majority of instances a moderate leucocytosis exists, this being especially marked in severe cases; I have never seen the number of cells go higher than thirteen thousand. On examining fixed preparations a diminution in the number of polymorphonuclear leucocytes is found, while there is a decided increase in the lymphocytes and eosinophiles. It is a remarkable fact that the severity of the general symptoms and the degree of anemia do not depend directly upon the number of parasites present in the intestine. In an instance of the disease in a child ten years of age where the number of red cells was 1,550,000, and the hemoglobin 10 per cent., only forty worms were expelled after treatment, and subsequent examinations showed that none were left in the intestinal tract. On the other hand, I recently saw a medical student who was in good health, comparatively speaking, who was not greatly anemic, and who was well developed, from whom there were obtained 960 worms. It is extremely difficult to say how long the disease may last, as the first symptoms come on insidiously, and it is even common for those who have had the disease some time to look upon themselves as being healthy, I have seen one case where the malady had probably existed for fourteen years, but in this instance repeated reinfection had probably occurred. We do not know how long the American hookworm may live, but it is said that the old world species attains an age of from five to eight years.

*Treatment.*—No malady so grave as this can be cured with such rapidity and certainty, and the time necessary to complete the treatment is only about twenty-four hours. Two drugs are said to act efficiently in killing the parasites—thymol and male fern—the latter of which I have never employed, but it should be given in the ordinary dose after previously preparing the patient in the same way as when thymol is to be administered. On the day before either of these drugs is to be given the patient should take a very light dinner, and no supper at all should be eaten. In the afternoon ten grains of calomel are administered, and if this should not act before bedtime it is well to give in addition either oil or some saline. During the night the bowels should move at least three times in order that the fecal matter as far as is possible may be removed from around the parasites; if the proper degree of purgation be not secured laxatives should be given on the following morning until several evacuations are produced, after which the thymol is administered. In those instances where the bowels act properly during the night the patient is given thirty grains of finely powdered thymol on awakening in the morning, and two hours later a similar dose is again exhibited. The sufferer eats no breakfast or dinner, and remains confined to bed during the day. Toward noon coffee or tea may be allowed, but beverages containing alcohol should be strictly interdicted for the reason that they dissolve the thymol and permit its absorption, and under such circumstances its poisonous effects may be produced. After the second dose of thymol is administered the stools are carefully preserved, and should be examined to determine if the parasites have been expelled. Late in the afternoon a dose of salts is given, following which the bulk of the worms are generally passed. The patient is allowed to eat his usual supper. It may be thought by the inexperienced that sixty grains of thymol within two hours is a rather large dose, but I have never seen any ill effects from it except in an instance where, as the result of taking some whisky an hour or so after the last dose of the drug was given, slowness of the pulse and slight delirium were produced. In the youngest patient that I have seen with this disease, who was a girl of ten years of age and extremely weak, absolutely no ill effect followed the administration of the quantity of the medicament above mentioned. Of course in young chlldren the dose should be less. Leichtenstern has even administered as much as two and a half drams of finely powdered thymol within the prescribed period, and saw no ill effects result. When the treatment is carried out as indicated I have never seen an instance where complete expulsion of the worms was not secured, but if the patient fails to follow the directions, particularly as regards not eating the day before, free purgation, and the taking of *sixty grains of finely powdered thymol*, this result may not be obtained. Following this treatment it is really unnecessary to do anything further, as from a number of observations I am convinced that tonics, such as strychnin, quinine, iron, arsenic, etc., exert no decided influence toward restoring the patient more rapidly to health, but of course there can be no harm in giving them. In recent cases, and in those that are not severe, recovery takes place rapidly, but on the other hand in instances where profound anemia exist, regeneration of the blood, and return of strength occur very slowly, a period of from three to six months being necessary in order for this to be brought about. Notwithstanding the fact that my results have been uniformly good, it is highly probable, as is the case in the old world, that occasionally no benefit will result from treatment, and that death from the disease itself, or some intercurrent malady, will sooner or later supervene.

The profession in the south should earnestly take up this matter, for, as I have said in a previous paper, in no other serious disease does the victim suffer so long, in no other condition is he for such a period a menace to those about him, and in no other malady of such gravity is the treatment so rapidly and surely successful.

As was stated in the beginning of this paper, most of my work upon this subject was done in southern Georgia and northern

Florida, where I was more particularly engaged in studying malaria. My observations were practically confined to the towns along the Georgia, Southern and Florida Railway, and I do not feel that I can close this paper without extending my thanks to the Hon. John I. Hall, General Counsel, for his kindness in requesting, and to Mr. William Checkley Shaw, Vice-President of this road, for his generosity in granting me free transportation over the line which he controls. This acknowledgment affords me a great deal of pleasure, as it is a rather unusual spectacle in our commercial times for men of affairs to exhibit such interest in scientific matters, an interest which I wish every one living along the line of this road could know of and appreciate.

## Figures and Tables

**Fig. 1. f1:**
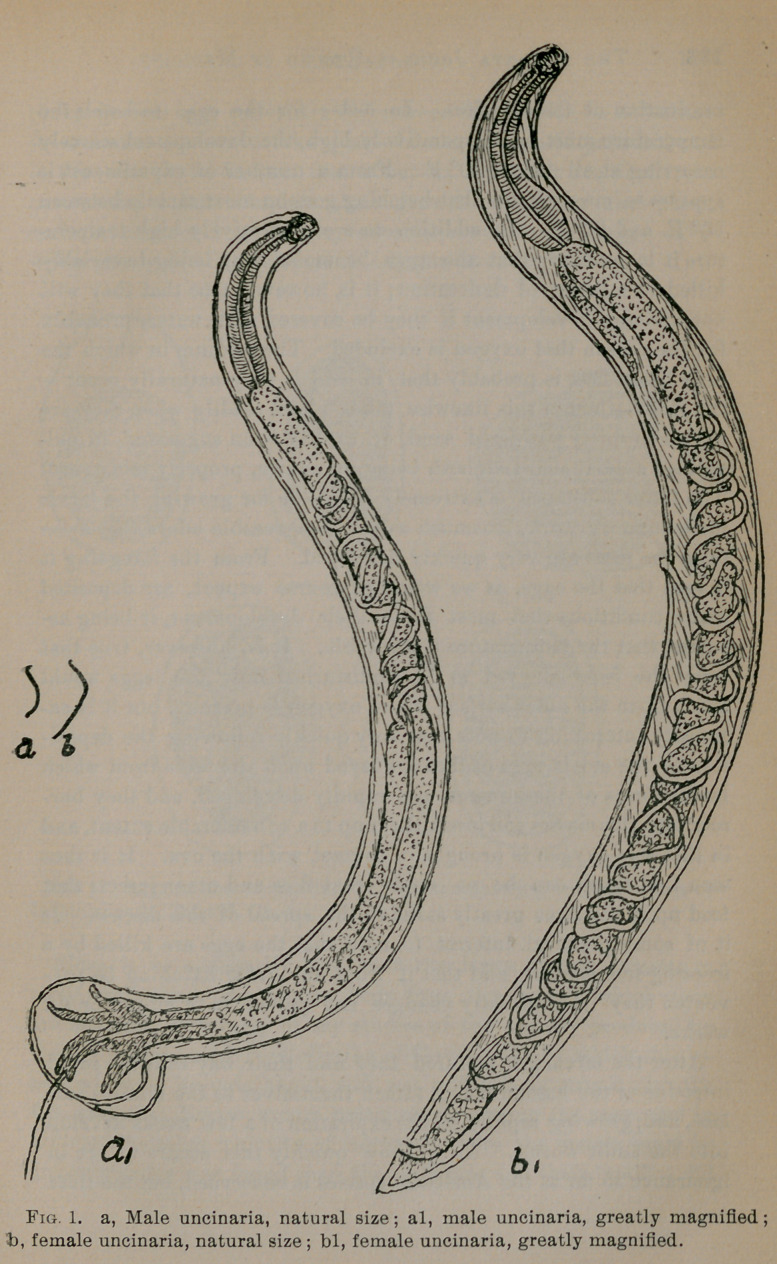


**Fig. 2. f2:**
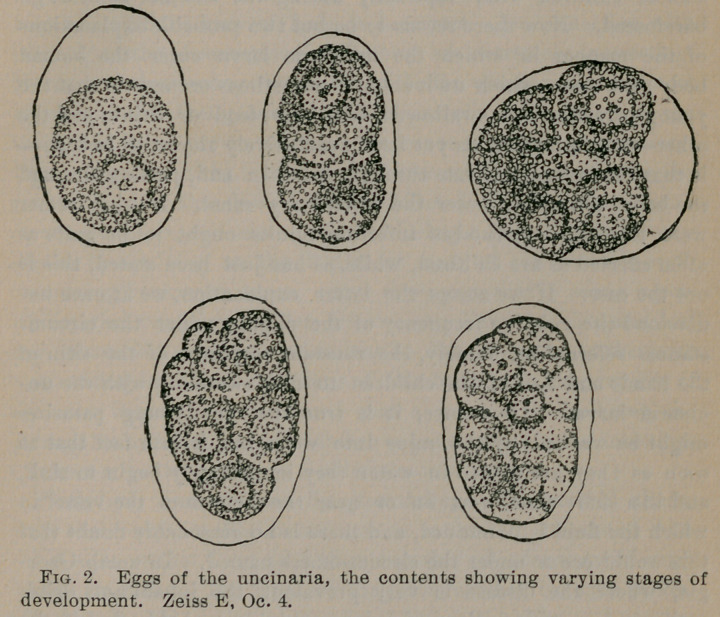


**Fig. 3. f3:**